# Lack of α8 integrin leads to morphological changes in renal mesangial cells, but not in vascular smooth muscle cells

**DOI:** 10.1186/1471-2121-11-102

**Published:** 2010-12-31

**Authors:** Ines Marek, Gudrun Volkert, Angelika Jahn, Fabian Fahlbusch, Christina Zürn, Zehra Özcan, Margarete Goppelt-Struebe, Karl F Hilgers, Wolfgang Rascher, Andrea Hartner

**Affiliations:** 1 Hospital for Children and Adolescents, Universität Erlangen-Nürnberg, Loschgestrasse 15, 91054 Erlangen, Germany; 2 Department of Nephrology and Hypertension, Universität Erlangen-Nürnberg, Loschgestrasse 8, 91054 Erlangen, Germany

## Abstract

**Background:**

Extracellular matrix receptors of the integrin family are known to regulate cell adhesion, shape and functions. The α8 integrin chain is expressed in glomerular mesangial cells and in vascular smooth muscle cells. Mice deficient for α8 integrin have structural alterations in glomeruli but not in renal arteries. For this reason we hypothesized that mesangial cells and vascular smooth muscle cells differ in their respective capacity to compensate for the lack of α8 integrin.

**Results:**

Wild type and α8 integrin-deficient mesangial cells varied markedly in cell morphology and expression or localization of cytoskeletal molecules. In α8 integrin-deficient mesangial cells α-smooth muscle actin and CTGF were downregulated. In contrast, there were no comparable differences between α8 integrin-deficient and wild type vascular smooth muscle cells. Expression patterns of integrins were altered in α8 integrin-deficient mesangial cells compared to wild type mesangial cells, displaying a prominent overexpression of α2 and α6 integrins, while expression patterns of the these integrins were not different between wild type and α8 integrin-deficient vascular smooth muscle cells, respectively. Cell proliferation was augmented in α8 integrin-deficient mesangial cells, but not in vascular smooth muscle cells, compared to wild type cells.

**Conclusions:**

Our findings suggest that α8 integrin deficiency has differential effects in mesangial cells and vascular smooth muscle cells. While the phenotype of vascular smooth muscle cells lacking α8 integrin is not altered, mesangial cells lacking α8 integrin differ considerably from wild type mesangial cells which might be a consequence of compensatory changes in the expression patterns of other integrins. This could result in glomerular changes in α8 integrin-deficient mice, while the vasculature is not affected in these mice.

## Background

Integrin family receptors mediate cell-cell or cell-matrix interactions. Integrins are heterodimers consisting of an α and a β subunit. At least 18 α and 8 β chains are known to date, which combine to 24 integrin receptors [1]. Most receptors recognize more than one ligand and each ligand is capable of binding several integrins, which leads to a wide variety of possible interactions [2]. Many β1 and β3 integrins are receptors for extracellular matrix molecules mediating not only adhesion of cells but also conveying signals which affect cytoskeletal architecture and thus cell morphology and differentiation (reviewed in [3-5]): In renal cells, signaling via integrins can alter the expression of cytoskeletal proteins [6] and the arrangement of cytoskeletal components, which is mediated via integrin linked kinase [7,8]. Several studies have suggested a regulatory role for integrins in the differentiation of epithelial cells [9], podocytes [6], mesangial cells [10] or fibroblasts [11]. Moreover, integrin signaling is involved in epithelial to mesenchymal transition, a phenomenon frequently seen in models of renal fibrotic disease [12,13].

The α8 integrin chain is expressed predominantly on mesenchymal cells, like vascular smooth muscle cells, certain fibroblast cells and glomerular mesangial cells, where it serves as a receptor for fibronectin, vitronectin, tenascin-C fragments, osteopontin and nephronectin, but not for collagens [14-17]. Moreover, a role for α8β1 integrin in migration, proliferation and survival of cells was described [18,19].

A few studies suggested that α8 integrin may be involved in cell differentiation processes: α8 integrin expression was reported to contribute to the maintenance of the smooth muscle cell differentiated phenotype, because downregulation of α8 integrin led to a severe reduction of α-smooth muscle actin expression and an increase in cell motility [20], while overexpression of α8 integrin had the opposite effects [21]. In fibroblasts, the downregulation of α8 integrin resulted in epithelialization, possibly due to induced WT-1 expression [22].

A role for a8 integrin was suggested in atherosclerotic [23] and fibrotic diseases [24,25]. Changes in the cytoskeletal architecture of cells could influence their ability to adhere and migrate. This might have an important impact on the progression of atherosclerotic or fibrotic diseases. On the other hand, we did not observe alterations of the smooth muscle cell layers of renal arteries and arterioles in α8 integrin-deficient mice, whereas the glomerular mesangium of these mice was clearly abnormal [26]. Thus, we hypothesized that mesangial cells and vascular smooth muscle cells might be differently affected by a lack of α8 integrin. We investigated if the properties of mesangial and vascular smooth muscle cells isolated from α8 integrin-deficient mice differ from their respective counterparts cultured from wild type controls.

## Results

**Figure 1:**
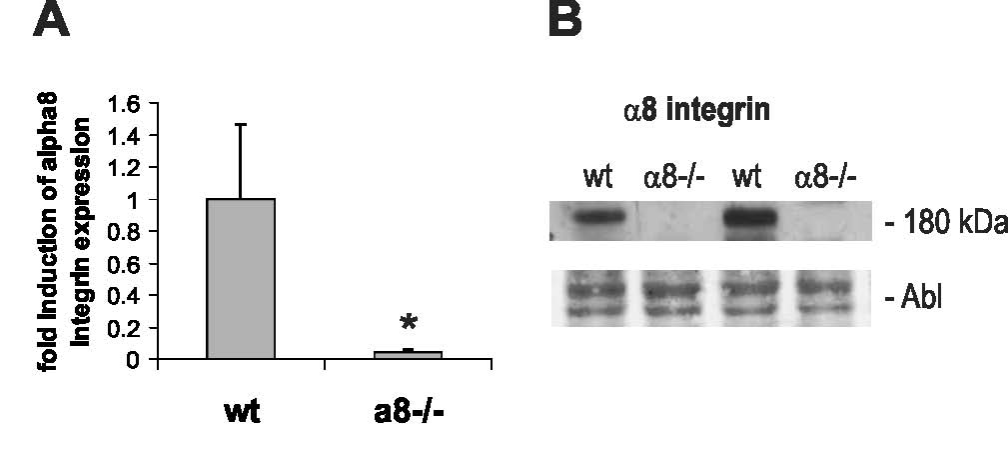
**Mesangial cell expression of α8 integrin in wild type (wt) and α8 integrin-deficient mesangial cells (α8-/-)**. A: Real-time RT-PCR analysis of α8 integrin expression in wt and α8-/- mesangial cells. B: Western blot analysis of α8 integrin protein in wt and α8-/- mesangial cells. Amido black (Abl) staining of the blot served as loading control. Results are representative for at least 3 similar experiments. Data are means ± sd. * p < 0.05 vs. wt.

Cultivated wild type and α8 integrin-deficient mouse mesangial cells (MCs) were tested for mRNA and protein expression of α8 integrin to confirm lack of α8 integrin expression in α8 integrin-deficient MCs and presence of α8 integrin in wild type cells. α8 integrin mRNA expression was readily detected in wild type MCs by real-time RT-PCR (Figure 1A), while α8 integrin mRNA expression was within background detection in α8 integrin-deficient MCs (Figure 1A). a8 integrin protein was detected in wild type MCs, but not in a8 integrin-deficient MCs by Western blot analysis (Figure 1B).

**Figure 2:**
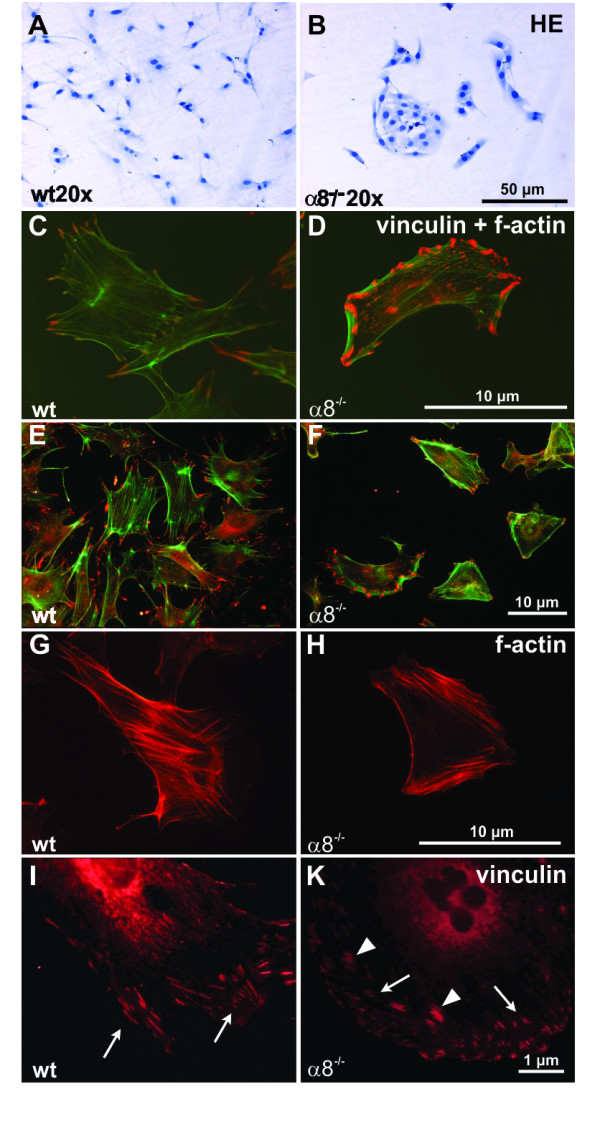
**Comparison of wild type (wt) and α8 integrin-deficient (α8-/-) mesangial cell morphology after hematoxylin stain (A+B), immunofluorescent double staining for f-actin in green and vinculin in red (high magnification C+D, low magnification E+F), immunofluorescent staining for f-actin alone (G+H) or immunofluorescent staining for vinculin alone (I+K)**. White arrows indicate focal contacts of the cells and white arrowheads indicate bundles of focal contacts in α8-/- mesangial cells.

**Figure 3:**
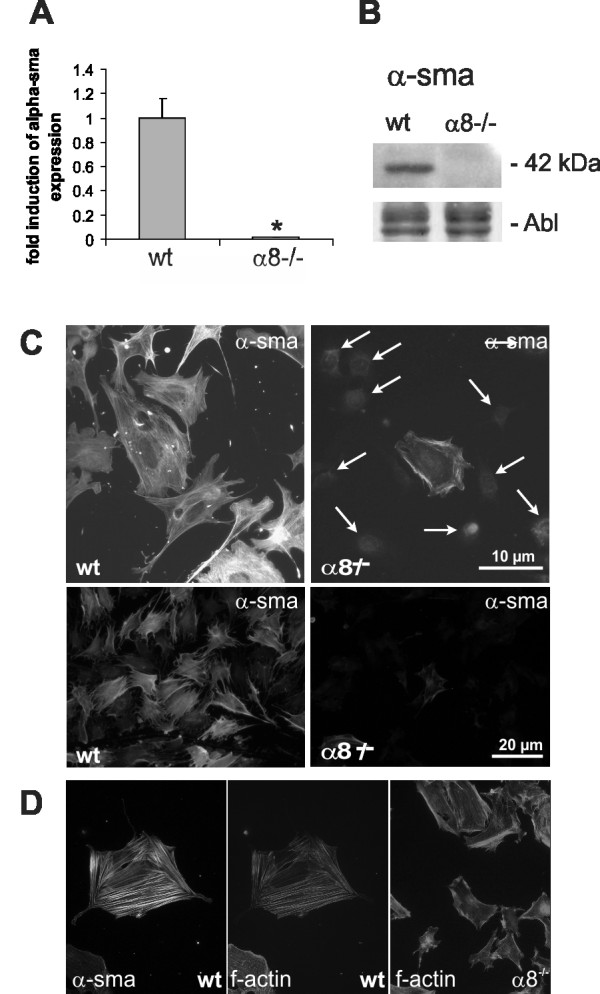
**Comparison of α-smooth muscle actin expression in wild type (wt) and α8 integrin-deficient (α8-/-) mesangial cells**. mRNA expression analysis by real-time RT-PCR analysis (A), representative Western blot analysis with amido black (Abl) staining as loading control (B) and immunofluorescent detection of α-smooth muscle actin (C). Arrows indicate cell nuclei of cells negative for α-smooth muscle actin. D: Doublestaining of wild type mesangial cells for α-smooth muscle actin (left) and f-actin (middle panel). Detection of f-actin in α8-/- mesangial cells (right). Results are representative for at least 3 similar experiments. Data are means ± sd. * p < 0.05 vs. wt.

Wild type MCs in culture grew in a typical mesenchymal pattern (Figure 2A), while the morphology of α8 integrin-deficient MCs was different with a more compact cell shape lacking long protrusions. Moreover, α8 integrin-deficient MCs were arranged in groups forming cell islets (Figure 2B). F-actin fibers were frequently arranged parallel to the cytoplasma membrane in α8 integrin-deficient MCs (Figure 2D, F and 2H), while in wild type MCs f-actin fibers were spanning the whole cell more irregularily (Figure 2C, E and 2G) after an attachment time of 24 hours. Focal contacts were shorter in α8 integrin-deficient MCs compared to wild type MCs and frequently arranged in bundles (Figure 2D and 2K). mRNA expression of α-smooth muscle actin was clearly detectable in wild type MCs but downregulated in α8 integrin-deficient MCs almost to background levels (Figure 3A). α-smooth muscle actin protein was barely detectable by Western blot analysis in α8 integrin-deficient MCs (Figure 3B). While wild type MCs α-smooth muscle actin stain was arranged in typical stress fibers, most α8 integrin-deficient MCs stained negative for α-smooth muscle actin, except for some occasional staining in short cortical fibers along the plasma membrane (Figure 3C). Double staining for α-smooth muscle actin and f-actin confirmed that in wild type MCs α-smooth muscle actin is a component of stress fibers (Figure 3D). In α8 integrin-deficient MCs f-actin staining is preserved despite the lack of α-smooth muscle actin immunoreactivity, arguing for a contribution of other types of actin to f-actin-positive fibers (Figure 3D).

**Figure 4:**
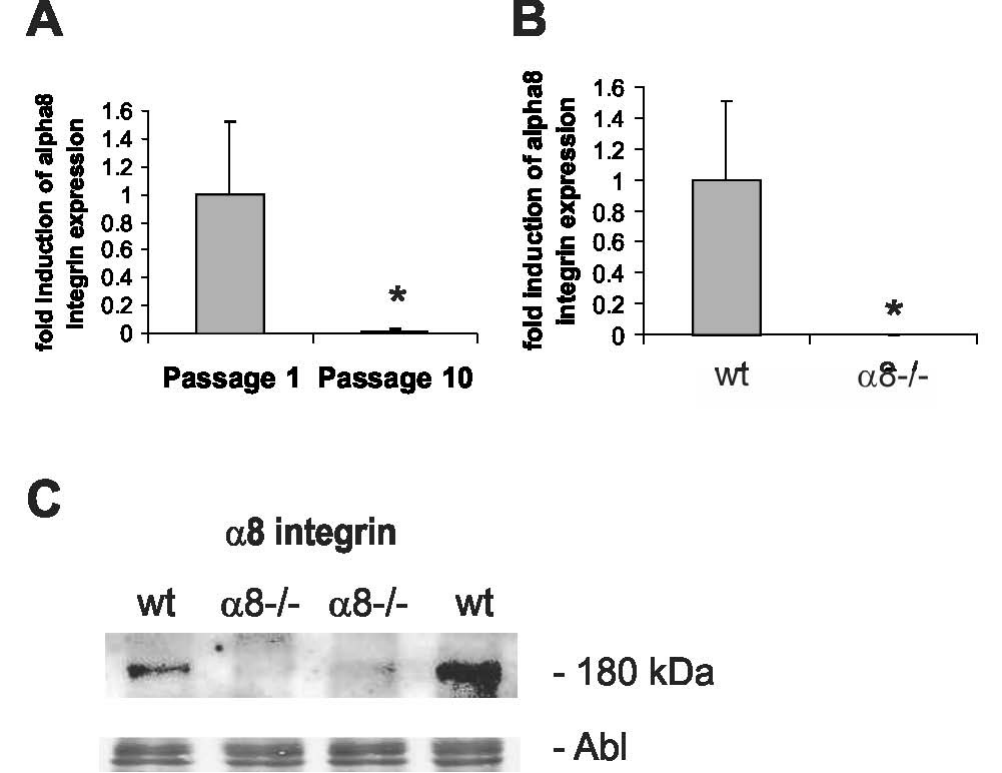
**Expression of α8 integrin in vascular smooth muscle cells**. Real-time RT-PCR analysis of α8 integrin expression in wild type (wt) vascular smooth muscle cells cultivated in passage 1 or 10 (A). Real-time RT-PCR analysis of α8 integrin expression in freshly isolated wt and α8-/- vascular smooth muscle cells (B). Western blot analysis of α8 integrin expression in freshly isolated wt and α8-/- vascular smooth muscle cells (C). Amido black (Abl) staining of the blot served as loading control. Results are representative for at least 3 similar experiments. Data are means ± sd. * p < 0.05 vs. freshly isolated cells or wt, respectively.

In contrast to MCs, vascular smooth muscle cells (VSMCs) downregulate α8 integrin expression after cell passaging in culture. In freshly isolated VSMCs of passage 1, α8 integrin expression was readily detectable by real-time RT-PCR, while in VSMCs after 10 passages, α8 integrin became nearly undetectable (Figure 4A). For this reason, we only used freshly isolated VSMCs in passage 1 for our further experiments. Lack of α8 integrin expression in α8 integrin-deficient VSMCs was confirmed by real-time RT-PCR (Figure 4B) and Western blot analysis (Figure 4C).

**Figure 5:**
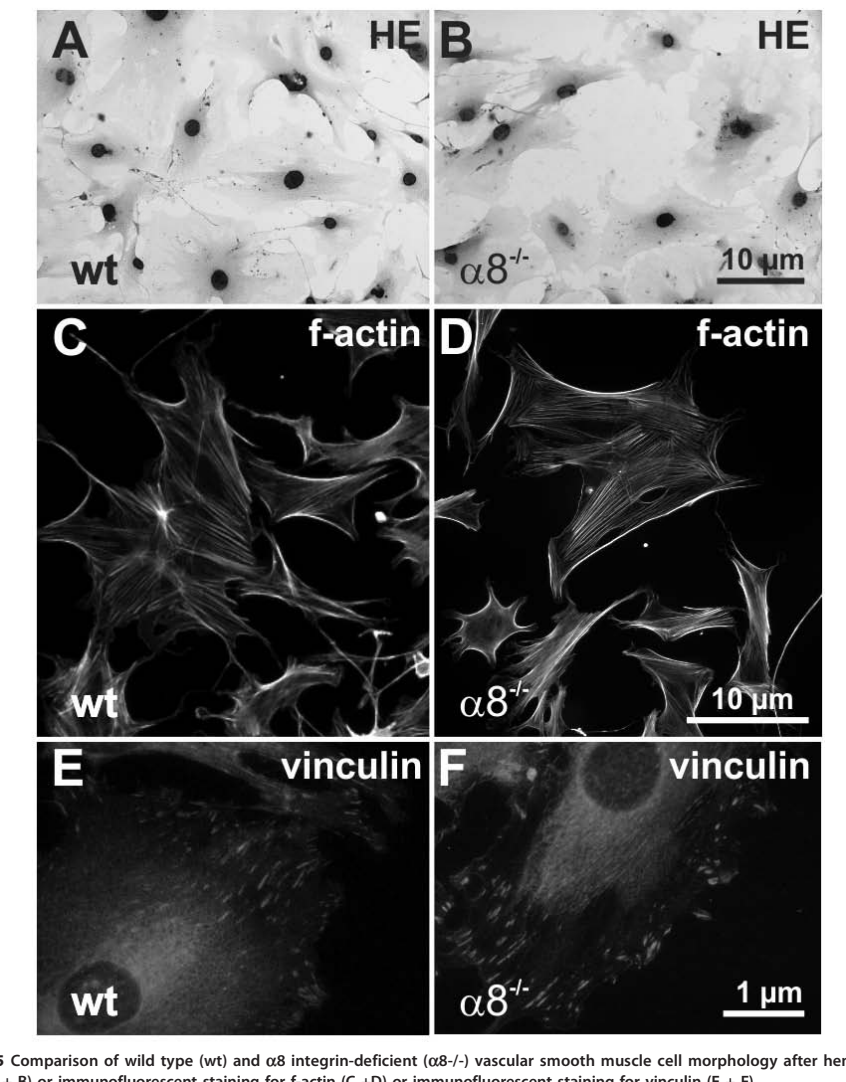
**Comparison of wild type (wt) and α8 integrin-deficient (α8-/-) vascular smooth muscle cell morphology after hematoxylin stain (A + B) or immunofluorescent staining for f-actin (C +D) or immunofluorescent staining for vinculin (E + F)**.

In contrast to our findings in MCs, cell morphology was not different in wild type and α8 integrin-deficient VSMCs (Figure 5A and 5B). Moreover, the distribution of f-actin fibers was not different in α8 integrin-deficient VSMCs compared to wild type VSMCs (Figure 5C and 5D) after 24 hours of attachment. Adherent α8 integrin-deficient VSMCs developed focal contacts, which were comparable to the focal contacts of wild type VSMCs (Figure 5E and 5F).

**Figure 6:**
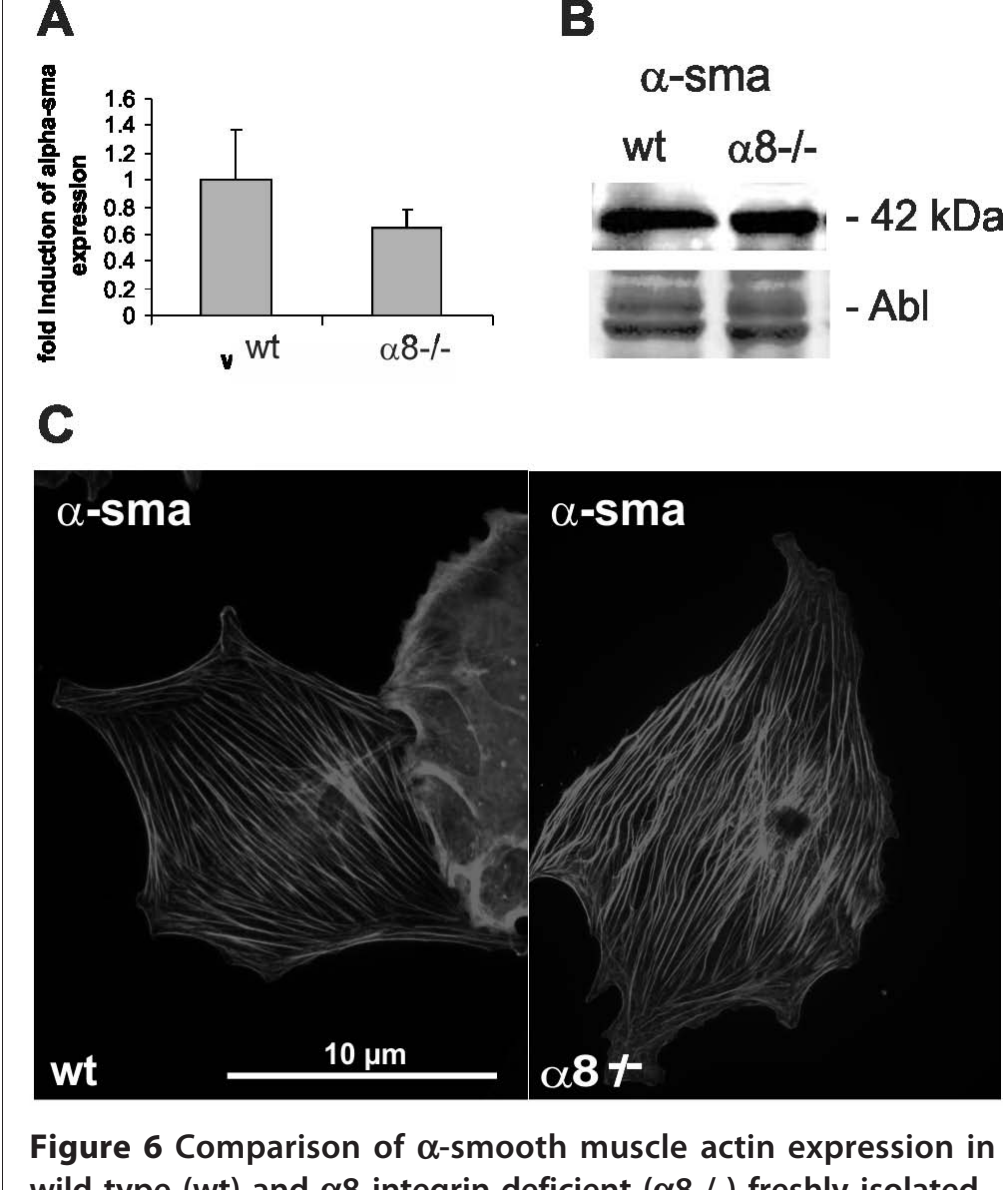
**Comparison of α-smooth muscle actin expression in wild type (wt) and α8 integrin-deficient (α8-/-) freshly isolated vascular smooth muscle cells**. α8 integrin expression by real-time RT-PCR analysis (A), Western blot analysis with amido black (Abl) staining as loading control. (B) and immunofluorescent detection (C) (x1000). Results are representative for at least 3 similar experiments.

Evaluation of α-smooth muscle actin expression revealed no significant differences between wild type and α8 integrin-deficient VSMCs (Figure 6A). Western blot analysis revealed that α-smooth muscle actin protein was abundant in wild type as well as α8 integrin-deficient VSMCs (Figure 6B). α-smooth muscle actin fibers were spanning the whole cell and were not reduced in α8 integrin-deficient VSMCs compared to wild types (Figure 6C).

**Figure 7:**
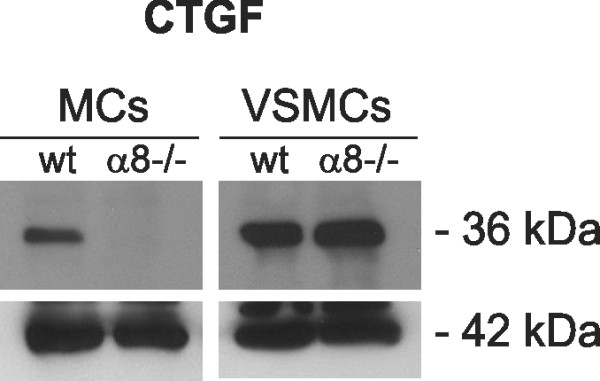
**Connective tissue growth factor (CTGF; 36 kDa) protein expression in wild type (wt) and α8 integrin-deficient (α8-/-) mesangial cells (MCs) and vascular smooth muscle cells (VSCMs)**. Staining for β-actin was used as a loading control. Results are representative for 3 similar experiments.

As reorganisation of the actin cytoskeleton can lead to changes in the expression of connective tissue growth factor (CTGF), we assessed protein expression levels of CTGF in wild type and α8 integrin-deficient MCs in comparison to VSMCs. In MCs, a lack of α8 integrin resulted in downregulation of the protein expression of CTGF (Figure 7), while in α8 integrin-deficient VSMCs CTGF expression was not reduced (Figure 7).

**Figure 8:**
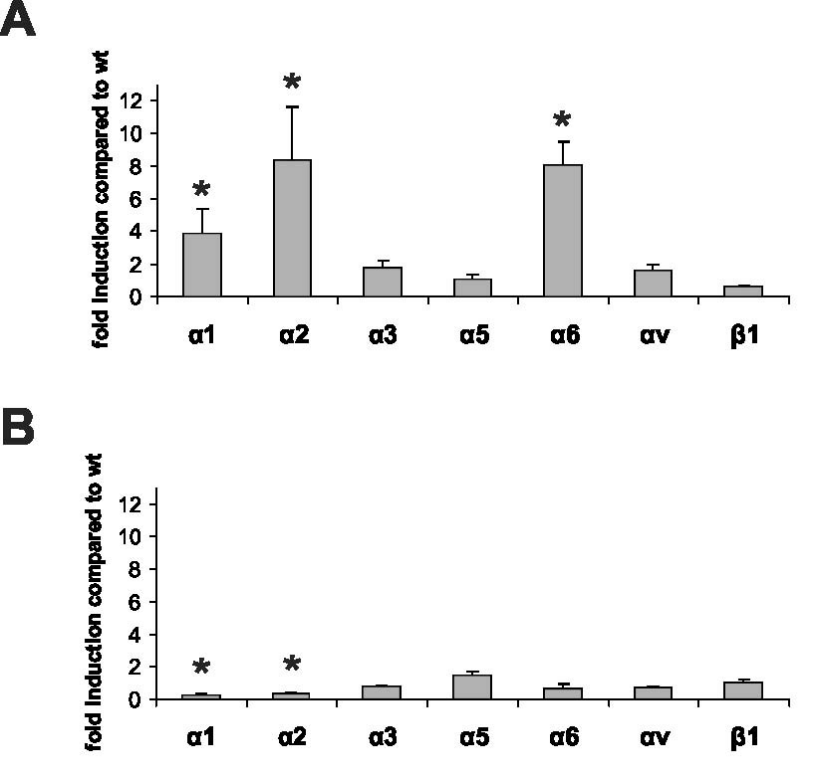
**Real-time RT-PCR analysis of integrin chain α1, α2, α3, α5, α6, αv and β1 expression profiles in wild type (wt) and α8 integrin-deficient (α8-/-) mesangial cells (A) and freshly isolated vascular smooth muscle cells (B)**. Results are representative for at least 3 similar experiments. Data are means ± sd. * p < 0.05 vs. wt.

We hypothesized that the differences in the effects of the lack of α8 integrin on cytoskeletal organization observed in MCs and VSMCs could be a consequence of a different regulation of other integrins in both cell types. Therefore, we compared expression patterns of several integrins in α8 integrin-deficient cells and wild type cells. In α8 integrin-deficient MCs, induction of integrin chains α1, and even more prominently of α2 and α6, was detected when compared to wild type MCs (Figure 8A). In contrast, none of the investigated integrin chains was induced in α8 integrin-deficient VSMCs (Figure 8B).

**Figure 9:**
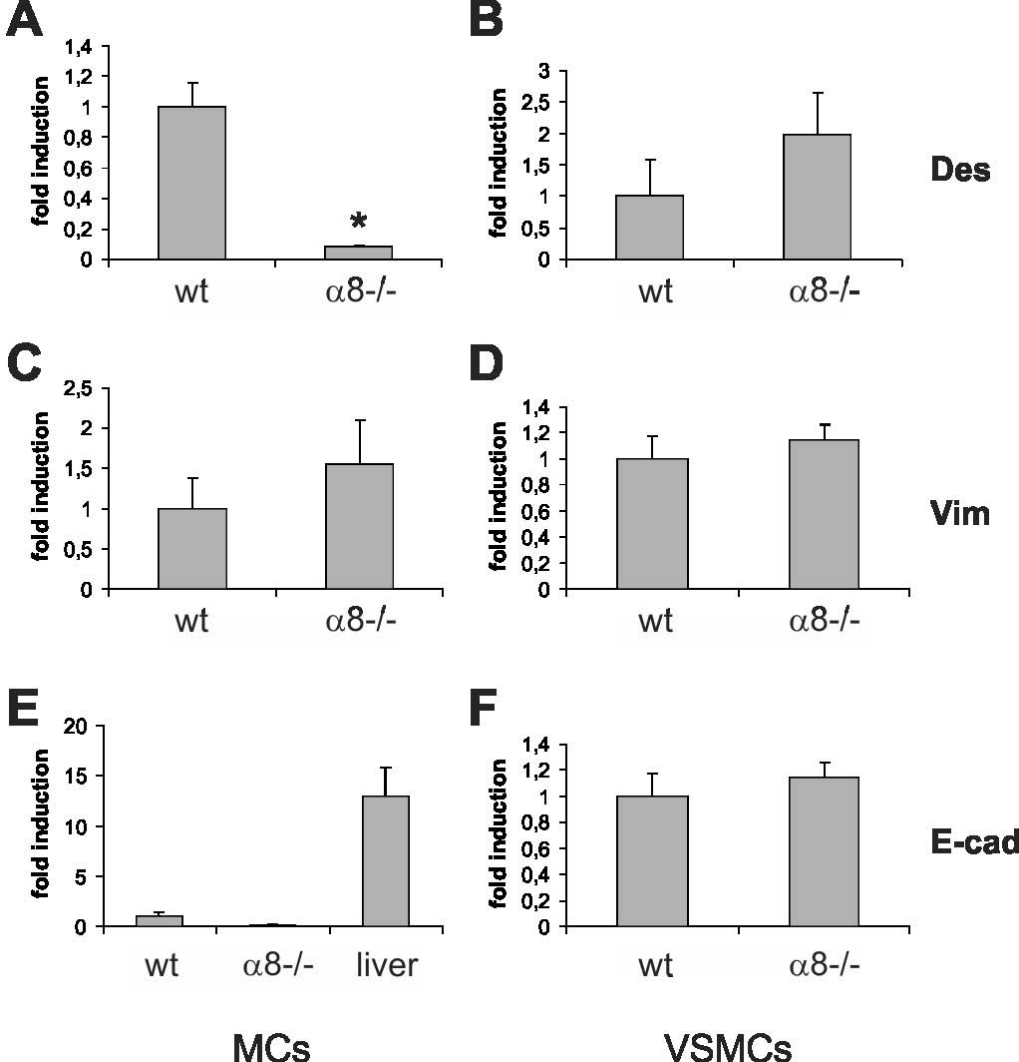
**Real-time RT-PCR analyses of desmin (Des), vimentin (Vim) and E-cadherin (E-cad) in wild type (wt) and α8 integrin-deficient (α8-/-) mesangial cells (MCs) and freshly isolated vascular smooth muscle cells (VSMCs)**. For E-cadherin expression in MCs, a positive control (liver cells) was used. Results are representative for at least 3 similar experiments. Data are means ± sd. * p < 0.05 vs. wt.

We then hypothesized that MCs lacking α8 integrin might downregulate not only α-smooth muscle actin but also additional mesenchymal markers. To analyze expression patterns of other mesenchymal markers, we performed real-time RT-PCR for vimentin and desmin and for the epithelial marker E-cadherin, because α6 integrins are known to upregulate E-cadherin-mediated adhesion [27]. In α8 integrin-deficient MCs the expression of desmin was significantly lower than in wild type MCs (Figure 9A), while in α8 integrin-deficient VSMCs desmin expression was not significantly different from desmin expression in wild type VSMCs (Figure 9B). On the other hand, vimentin expression was not affected by the lack of α8 integrin, in none of the cell types (Figure 9C and 9D). E-cadherin expression was barely above detection level in both wild type and α8 integrin-deficient MCs, compared to its expression in liver cells used as positive control (Figure 9E). In VSMCs, expression of E-cadherin was not different in wild type and α8 integrin-deficient cells (Figure 9F).

**Figure 10:**
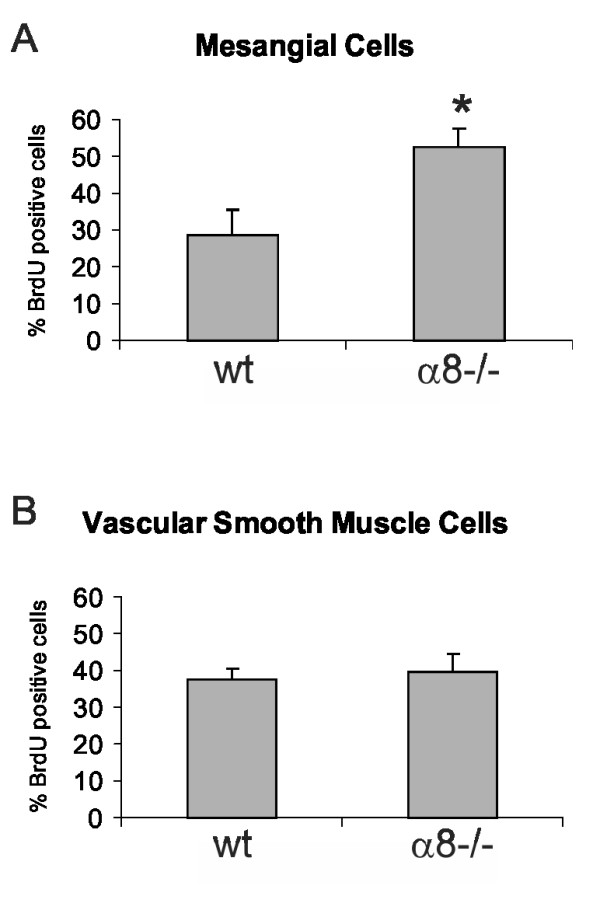
**Cell proliferation of wild type (wt) and α8 integrin-deficient (α8-/-) mesangial cells (A) and vascular smooth muscle cells (B) in response to stimulation with 10% FCS**. Results are representative for at least 3 similar experiments. Data are means ± sd. * p < 0.05 vs. wt.

To clarify if these differences of the properties of α8 integrin-deficient MCs and VSMCs have functional consequences, we performed proliferation assays. Our results show that α8 integrin-deficient MCs and VSMCs differ in their growth response to fetal calf serum when grown on fibronectin, a ligand for α8 integrin (Figure 10). While stimulation of α8 integrin-deficient MCs leads to significantly more proliferation than stimulation of wild type MCs, proliferation of wild type and α8 integrin-deficient VSMCs was not different.

## Discussion

Taken together, a8 integrin-deficient MCs differed from wild type MCs with regard to morphology, cytoskeletal architecture and proliferative capacity, while a8 integrin-deficient VSMCs did not differ from wild type VSMCs. This is in keeping with our previous in vivo findings suggesting changes in the glomerular mesangium but not in the media of renal arteries in a8 integrin-deficient mice [26], although in normal mice both structures contain mesenchymal cells expressing a8 integrin. a8 integrin-deficient MCs downregulated expression of a-smooth muscle actin and desmin, but not vimentin, while expression of these mesenchymal proteins was not altered in a8 integrin-deficient VSMCs. a8 integrin-deficient MCs had higher expression levels of integrin chains a1, a2 and a6 compared to wild type MCs. Similar differences were not detected between a8 integrin-deficient and wild type VSMCs. Moreover, increased proliferation rates due to a lack of a8 integrin were only detected in MCs, not in VSMCs.

Several studies show that integrins can contribute to cell differentiation and to the maintenance of the phenotype of the cell via outside-in signaling from the surrounding matrix to the cytoskeleton and small adapter molecules inside the cell [4,28]. Many integrins use a signaling pathway involving the β1 integrin chain and integrin linked kinase to regulate the cytoskeletal architecture of the cell [29]. Moreover, integrins can alter the organization of the actin cytoskeleton via proteins of the rho family, which also regulate CTGF [30]. CTGF, besides having profibrotic function, can act as a mediator of growth arrest [31]. In MCs, disassembly of actin stress fibers with an inhibitor of rho family proteins resulted in inhibition of CTGF expression [32]. We could show that MCs lacking a8 integrin rearrange their actin cytoskeleton and downregulate CTGF.

Changes in the cytoskeletal architecture can alter cell adhesion and motility [33]. In a previous study, we showed that compared to wild type MCs, a8 integrin-deficient MCs adhered weaker to fibronectin and vitronectin, two ligands for a8 integrin, but adhered more easily on collagens, which are not ligands for a8 integrin [18]. On the other hand, a8 integrin-deficient MCs migrated more easily on fibronectin or vitronectin than wild type cells [18]. These results support the notion that α8 integrin could serve as an anti-migratory integrin, keeping MCs resting at their native location. Firm adhesion, as mediated by α8 integrin, inhibits migration in many cell types [34]. Thus the decreased ability of α8 integrin-deficient MCs to adhere to fibronectin or vitronectin could contribute to the increased ability of these cells to migrate. Given these differences in migratory abilities, we hypothesized in the present study that wild type and α8 integrin-deficient MCs also differ in their cytoskeletal architecture and general morphology.

Downregulation of a-smooth muscle actin expression in a8 integrin-deficient MCs leads to a reduction in a-smooth muscle actin containing stress fibers and consequently to a reduction in firm adhesion. This in turn seems to lead to increased cell motility of a8 integrin-deficient MCs. Similar observations were made in VSMCs after siRNA knockdown of a8 integrin expression [20]: Treatment with a8 integrin siRNA reduced expression of a-smooth muscle actin and increased cell migration, which is in contrast to our findings in a8 integrin-deficient VSMCs, where both the a8 integrin-deficient and the wild type genotype expressed a-smooth muscle actin in comparable amounts. The reasons for the discrepancy of the results of the studies in VSMCs after blockade of a8 integrin expression with siRNA and in a8 integrin-deficient VSMCs are unclear at present. Our results regarding a-smooth muscle actin expression in a8 integrin-deficient MCs are reminiscent of the findings of Zaghram et al. [20] after siRNA blockade of a8 integrin in VSMCs. We therefore wanted to investigate the differences in a8 integrin-deficient MCs and a8 integrin-deficient VSMCs: A compensatory increase of integrin chains a1, a2 and a6 was detected in a8 integrin-deficient MCs. A similar increase of integrin expression was not found in a8 integrin-deficient VSMCs. Thus, it seems possible that changes in the cytoskeletal architecture and a-smooth muscle actin expression in a8 integrin-deficient MCs is not a direct consequence of the lack of a8 integrin, but more likely due to the induction of other integrin chains. a6 integrin is usually not expressed in MCs, but is an integrin characteristic of epithelial cells, while a8 integrin is a typical mesenchymal integrin [35,36]. During kidney development, downregulation of a8 integrin, possibly by WT-1 [22], results in epithelialization of mesenchymal cells and in the formation of tubular structures [37]. For this reason, we tested if a8 integrin-deficient MCs exhibit reduced expression of other mesenchymal markers or increased expression of a typical epithelial marker, widely used in the detection of epithelial-mesenchymal transition [38,39]. A reduction of desmin expression was readily detected, but vimentin expression was not reduced and E-cadherin expression was very low in a8 integrin-deficient MCs. These findings argue against the hypothesis that lack of a8 integrin, along with increased expression of a1, a2 and a6 integrins, leads to an epithelialization of MCs, but more likely might result in dedifferentiation of MCs. Why a8 integrin-deficient MCs undergo these changes in integrin expression and cytoskeletal architecture, while a8 integrin-deficient VSMCs do not, remains unclear. Discrepancies in the differentiation status might influence the ability of cells to dedifferentiate more easily than others. MCs and VSMCs might also use distinct transcriptional mechanisms, like it was described for smooth muscle cell and myofibroblast a-smooth muscle actin expression [40]. Moreover, no explanation exists to date as to why VSMCs after blockade of a8 integrin with siRNA behave differently from a8 integrin-deficient VSMCs regarding a-smooth muscle actin expression and cytoskeletal rearrangements. As shown by Zargham et al. [20], blockade of a8 integrin with siRNA results in a dysregulation of the expression of other integrins, like an increased expression of the a2, a5 and av chains, or reduced expression of the a1 chain. In our isolations of a8 integrin-deficient VSMCs we did not observe significant increases in the expression of the a2, a5 and av chains, while the expression of the a1 chain indeed was reduced. One has to be aware that acute blockade of a8 integrin with siRNA in VSMCs might not be consistent with a genetic knockdown of a8, which is more comparable to a chronic deficiency from the time of VSMC differentiation on. As a consequence, many regulatory pathways might differ in the two cell types. Moreover, the findings with blockade of a8 integrin with siRNA was obtained in rat VSMCs [20], while our data are derived from mouse VSMCs. Species differences might exist with regard to VSMC biology.

Finally, differences in the properties of MCs and VSMCc lacking a8 integrin were detected regarding cell growth. While a8 integrin-deficient MCs had significantly increased proliferation rates on ligands for a8 integrin compared to wild type MCs [18], wild type and α8 integrin-deficient VSMCs showed a comparable growth response after stimulation. Thus it is conceivable that the cytoskeletal and matrix receptor changes in α8 integrin-deficient MCs may result in changes in proliferative capacities of these cells. Both α2 and α6 integrin chains, which are upregulated in α8 integrin-deficient MCs, can promote cell proliferation [41,42]. On the other hand, increased proliferation rates in MCs lacking a8 integrin might be a consequence of rho-mediated disruption of actin stress fibers, leading to increased expression of CTGF, which was described to act anti-proliferative [31].

## Conclusion

A lack of a8 integrin appears to be of little consequence in VSMCs whereas the deficiency of this integrin has profound effects on the MC phenotype. The different abilities of both cell types to induce other integrin chains might well result in different phosphorylation patterns of kinases involved in integrin signaling, which could lead to a different activation of signaling cascades, causing differences in cytoskeletal characteristics and proliferation capacities.

## Methods

### Cultivation of mouse mesangial cells and vascular smooth muscle cells

Cells used in this study were obtained from organs of wild type or α8 integrin-deficient mice (obtained from U. Müller, Basel). Animal caretaking was performed according to the guidelines of the American Physiological Society and approved by local government authorities. Mesangial cells (MCs) were isolated from kidneys by the sieving method [43] using 63, 75 and 38 μm grid sieves. Cultured wild type and α8 integrin-deficient MCs were characterized as described [18]. MCs were grown in Dulbecco's modified Eagle's Medium (DMEM; PAA Laboratories GmbH, Linz, Austria) containing 10% FCS, 5 μg/ml insulin, 5 μg/ml plasmocin (TEBU, Frankfurt, Germany) and 2 mM L-glutamine (Sigma, Deisenhofen, Germany) in a 95% air - 5% CO_2 _humified atmosphere at 37°C. MCs were used for experiments in passages 5-10.

Vascular smooth muscle cells (VSMCs) were isolated from mouse aorta similar as described by Strehlow et al. [44] for rat vascular smooth muscle cells. Briefly, the aortas were excised, washed in phosphate-buffered saline with 1% penicillin-streptomycin and fat was removed with a fine forceps. The aortas were then incubated in DMEM containing 1 mg/ml collagenase type I (Sigma), 0.3 mg/ml elastase (Serva, Heidelberg, Germany) and 0.3 mg/ml trypsin inhibitor type II (Sigma) for 15 to 20 minutes at 37°C. The aorta was washed and the adventitia was stripped with fine forceps. The vessels were incised longitudinally and the endothelial cells were gently scraped off. The aortas were then minced with scissors and transferred to reaction tubes containing the same enzymatic solution as described above, incubated in 37°C for 60 to 90 minutes until 90% of the cells were dispersed under the microscope. The cells were centrifuged at 5000 rpm for 2 minutes, then resuspended in 3 ml DMEM with 20% fetal calf serum (FCS), 2% penicillin-streptomycin, and cultured in plates or flasks in a 95% air - 5% CO_2 _humidified atmosphere at 37°C for experiments. Cultured cells were verified to be VSMCs by immunostaining with anti-smooth muscle actin antibody (Sigma).

For detection of cytoskeletal components, MCs and VSMCs were allowed to attach on culture slides coated with 10 μg/ml fibronectin for 24 hours.

### Isolation of mRNA and Real-time PCR

To evaluate mRNA expression levels, total RNA was obtained from harvested cells by extraction with RNeasy^® ^Mini columns (Quiagen, Hilden, Germany). First-strand cDNA was synthesized with TaqMan reverse transcription reagents (Applied Biosystems, Weiterstadt, Germany) using random hexamers as primers. Final RNA concentration in the reaction mixture was adjusted to 0.1 ng/μL. Reactions without Multiscribe reverse transcriptase were used as negative controls for genomic DNA contamination. PCR was performed with an ABI PRISM 7000 Sequence Detector System and SYBR Green or TaqMan reagents (Applied Biosystems) according to the manufacturer's instructions. The relative amount of the specific mRNA was normalized with respect to 18 S rRNA. Primers used for amplification are listed in Table 1. For detection of E-cadherin mRNA, a TaqMan probe was used: 5'-GTC ACA GAC CCC ACG ACC AAT GAT-3'. All samples were run in triplicates.

**Table 1:** Primer pairs for Sybr green analysis

	forward	reverse
α-smooth muscle actin	5'-CCC TGA AGA GCA TCC GAC AC-3'	5'-GCC TTA GGG TTC AGT GGT GC-3'
α1 integrin	5'-CCA GTC AGC AGC TTC GTT TGA-3'	5'-TTC CAG TCA TAG GCT CCC ACA G-3'
α2 integrin	5'-TGA CCA GGT TCT GCA GGA TAG A-3'	5'-AGT AGA AAT TGC AGC CAC AGA GTA AC-3'
α3 integrin	5'-AGG CAC AGG CTA TGG AGA ATC A-3'	5'-CGC ACT CTT TCT GGA AGT GGA C-3'
α5 integrin	5'-TCG GAG CAA CAG TTC GGG-3'	5'-GTG GAG CAC ATG CCA AGA TG-3'
α6 integrin	5'-TCC CCG ACT GGC ATA ATT ACC-3'	5'-CGA TGT CCC CTC GAG AAC C-3'
α8 integrin	5'-TCA AGG CGA GGA ACA GCA A-3'	5'-CCT TGG GAA CCC GAT GGT-3'
αv integrin	5'-GGA GCT TTT GGT GTG GAT CG-3'	5'-GAC AAC GGG TCT GGC TCT GTA-3'
β1 integrin	5'-TGG CAA CAA TGA AGC TAT CGT G-3'	5'-GTA GGA CAG TCT GGA GTC TCC ACA-3'
desmin	5'-GTG AAG ATG GCC TTG GAT GT-3'	5'-TTG AGA GCA GAG AAG GTC TGG-3'
vimentin	5'-ACG ATC TCA CCC TCA GGG CT-3'	5'-GGG TCG CTG AGT CAG TGG AT-3'
e-cadherin	5'-AAG TGA CCG ATG ATG ATG CC-3'	5'-CTT CAT TCA CGT CTA CCA CGT-3'
18S	5'-TTG ATT AAG TCC CTG CCC TTT GT-3'	5'-CGA TCC GAG GGC CTC ACT A-3'

### Western Blot Analysis

Protein concentration of cell lysates was determined using a protein assay kit (Pierce, Rockford, IL). Protein samples containing 30 μg total protein were denatured by boiling for five minutes and separated on a 8% denaturing SDS-PAGE gel. After electrophoresis, the gels were electroblotted onto PVDF membranes (Pall Filtron, Karlstein, Germany), blocked with 5% horse serum/TBS/0.1% Tween 20 for 2 hours and incubated with the primary antibody overnight. Immunoreactivity was visualized with a secondary horseradish peroxidase-conjugated anti-rabbit IgG antibody or anti-mouse IgG antibody (both from Santa Cruz Biotechnology, Heidelberg, Germany), using the ECL system according to the manufacturer's instructions (Amersham, Braunschweig, Germany).

### Immunocytochemistry

MCs and VSMCs were seeded on glass 8-well chamber slides blocked with 2% BSA. Cells were allowed to adhere for 24 h. Then, supernates were removed, adherent cells were rinsed 3× with PBS and fixed in 3% paraformaldehyde for 20 min. After blockade of free aldehyde groups with 50 mM ammonium chloride, cells were permeabilized by 1% Triton X-100 and nonspecific binding was blocked using 100% FCS. Cells were incubated with the primary antibodies overnight, followed by a CY3-labelled goat anti-rabbit or anti-mouse immunoglobulin G (Dianova) as secondary antibody and embedding in Tris-buffered Mowiol, pH 8,6 (Hoechst). F-actin was visualized with phalloidin from Molecular Probes (Leiden, The Netherlands).

### Antibodies

The rabbit polyclonal antiserum to α8 integrin was kindly provided by Dr. Ulrich Muller, San Diego and used at a dilution of 1:200 as described before [43]. A polyclonal antibody to CTGF (Santa Cruz Biotechnology) was used in a dilution of 1:1000. A monoclonal antibody to smooth muscle actin (DAKO Diagnostika, Hamburg, Germany) was used at a dilution of 1:50 for immunocytochemistry or 1:1000 for Western blot analysis. A polyclonal antibody to vinculin (Santa Cruz Biotechnology) was used in a dilution of 1:500 for immunocytochemistry.

### Determination of cell proliferation

To assess cell growth, a 5-bromo-2'-deoxy-uridine (BrdU) incorporation assay into cellular DNA was performed using a BrdU labeling and detection kit (#1299964; Roche Mannheim, Germany). Cells were washed two times with PBS and serum-starved for 72 hours in medium containing 0.1% FCS. After trypsinating and washing they were seeded into culture slides (Falcon, HTS; Becton Dickinson, Heidelberg, Germany) which had been coated with 10 μg/ml fibronectin and blocked with 2% BSA. After a 12-hour resting period allowing the cells to attach to the matrix, they were incubated with medium containing 10% FCS for 48 hours. For the last two hours of incubation, BrdU was added. Cells were then fixed with 70% ethanol (in 50 μM glycine buffer; pH 2.0) and processed following the manufacturer's instructions. Incorporated BrdU was detected by an alkaline phosphatase-conjugated secondary antibody reacting with an NBT/X-phosphate substrate. Cells were counterstained with hematoxylin. Nuclei with a positive staining for BrdU were counted. Results shown are representative for at least three independent experiments.

### Statistical analyses

A t-test was used to test significance of differences between groups. A P-value <0.05 was considered significant. The procedures were carried out using SPSS software (SPSS Inc., Chicago, USA). Values are displayed as means ± standard deviation (SD).

## List of Abbreviations

CTGF: connective tissue growth factor; MCs: mesangial cells; VSMCs: vascular smooth muscle cells

## Authors' contributions

IM, GV, AJ and ZO carried out the cell culture experiments, CZ and FF analysed the data and helped drafting the manuscript, KFH performed the statistical analyses, MGS, WR and AH participated in the design and coordination of the study, AH drafted the manuscript. All authors have read and approved the final manuscript.
